# RAEdb: a database of enhancers identified by high-throughput reporter assays

**DOI:** 10.1093/database/bay140

**Published:** 2019-01-08

**Authors:** Zena Cai, Ya Cui, Zhiying Tan, Gaihua Zhang, Zhongyang Tan, Xinlei Zhang, Yousong Peng

**Affiliations:** 1 College of Biology, Hunan University, Changsha, China; 2 Key Laboratory of RNA Biology, Institute of Biophysics, Chinese Academy of Sciences, Beijing, China; 3 University of the Chinese Academy of Sciences, Beijing, China; 4 College of Computer Science and Electronic Engineering, Hunan University, Changsha, China; 5 College of Life Sciences, Hunan Normal University, Changsha, China; 6 Suzhou Geneworks Biotechnology Co., Ltd., Suzhou, China

## Abstract

High-throughput reporter assays have been recently developed to directly and quantitatively assess enhancer activity for thousands of regulatory elements. However, there is still no database to collect these enhancers. We developed RAEdb, the first database to collect enhancers identified by high-throughput reporter assays. RAEdb includes 538 320 enhancers derived from eight studies, most of which were from six human cell lines. An activity score was assigned to each enhancer based on reporter assays. Based on these enhancers, 7658 epromoters (promoters with enhancer activity) were identified and stored in the database. RAEdb provides two ways of searches: the first is to search studies by species and cell line; the other is to search enhancers or epromoters by position, activity score, sequence and gene. RAEdb also provides a genome browser to query, visualize and compare enhancers. All data in RAEdb is freely available for download.

## Introduction

The enhancer is a short (50~1500 bps) region of DNA sequence that recruits transcription factors to regulate the transcription of target genes in a cell-type-specific manner (1). Enhancers play a central role in regulating a wide range of important biological functions and processes, such as embryogenesis, development and homeostasis (1–3). They were reported to be associated with many human complex diseases (4). There are several databases collecting enhancers, including EnhancerAtlas (5), VISTA Enhancer Browser (6), FANTOM5 (7), DENdb (8), dbSUPER (9) and SEA (10). Although millions of enhancers are collected in these databases, lots of enhancers may be `false positives’ because most of them were inferred by indirect methods that require further reporter assays to determine enhancer activity (11, 12). Moreover, most enhancers in previous databases lack of quantitative activity scores (11, 12), which hinders computational modeling of gene regulations.

In recent years, high-throughput reporter assays including STARR-seq (self-transcribing active regulatory region sequencing) (11, 12) and MPRA (massively parallel reporter assay) (13) have been developed to directly and quantitatively assess enhancer activity for thousands of regulatory elements. For example, Arnold *et al*. (11) identified thousands of cell-type-specific enhancers across a broad continuum of strengths and created a genome-wide quantitative enhancer map in Drosophila by STARR-seq. Despite of widespread applications of these new methods in identifying and characterizing enhancers, there is still a lack of database collecting these enhancers. Herein, we present RAEdb, the first database for hosting, analyzing and visualizing enhancers identified by high-throughput reporter assays. Besides the enhancers, thousands of epromoters were also inferred based on these enhancers, and were stored in the database.

**Figure 1:**
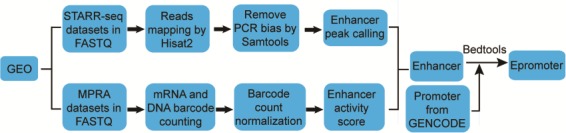
Workflow for enhancer and epromoter identification.

## Materials and methods

### Data sources

STARR-seq and MPRA data were collected from NCBI Gene Expression Omnibus (GEO) database (14) and the European Nucleotide Archive (15). We used a set of keywords (`STARR-seq’, `STARRseq’, `Self-transcribing active regulatory region sequencing’, ‘MPRA’ and `Massively parallel reporter assay’) to search these databases and screened the datasets manually. After manual check, four studies were collected for both STARR-seq and MPRA, respectively. As a comparison, several H3K27ac and H3K4me1 ChIP-seq datasets in some common cell lines were also downloaded from the ENCODE Consortium (16), and were displayed on genome browser derived from UCSC Genome Browser database (17).

### Enhancer peak calling from STARR-seq data

The workflow for calling enhancer peak from STARR-seq data were shown in Figure 1. Sequencing reads were aligned to human genome hg38 by Hisat2 (18). To exclude potential PCR amplification bias, fragments (inferred from paired-end reads) that have the same start and end positions were collapsed into distinct fragments by SAMtools (19). An enhancer peak is called when there is significant enrichment of fragments from one region in output library than the representation of that region in input library based on Poisson distribution using MACS2 (false discovery rate < 0.05) (20). The genome coverage of the plasmid library was used as input when calculating the enrichment of STARR-seq reads (11). We took the enrichment score reported by MACS2 as enhancer activity.

### Enhancer identification from MPRA data

The workflow for identifying enhancers from MPRA data was adapted from previous studies (21, 22), which was shown in Figure 1. To infer the barcode copy numbers generated for each sample, all sequence reads were examined, regardless of their quality scores. If the first N (N equal to the length of barcode) nucleotides of a read perfectly matched any of the barcode, this was counted as one occurrence of that barcode. The mRNA and plasmid counts for each barcode were then normalized by the size of cDNA and plasmid library, respectively, to facilitate comparing counts across samples with different sequencing depths. The library size was calculated as the total number of reads in each sample. For samples in multiple independent replications, the median of normalized barcode numbers were used. Finally, a ratio of mRNA to plasmid barcode counts was calculated to measure enhancer activity of the sequence linked to the barcode. If multiple different barcodes were linked to the same sequence, the average ratio was used. Only the sequences with the ratio greater than 1 were considered as enhancers.

### Epromoter identification

We defined the region of 500 bps upstream and downstream of the Transcription Start Site as promoter based on the Ensembl Genome Browser (23). The promoters overlapping with one or more enhancers were considered as epromoters (promoters with enhancer activity) with the help of bedtools (version 2.27.0) (24, 25).

### Implementation

An open source platform, LAMP (Linux, Apache, MySQL and PHP), is used to implement RAEdb database. The JavaScript Object Notation format is employed to implement the communication between the client-side and server-side layer. The JavaScript library JQuery and the web framework bootstrap were employed for producing dynamic and interactive data visualization in the web interface. In addition, DataTable library was applied to construct tables in web pages.

In addition, we integrated UCSC Genome Browser (17) in our database for visualizing enhancers. The annotation of genes and references are hosted in the genome browser.

## Results

### Data summary

**Figure 2:**
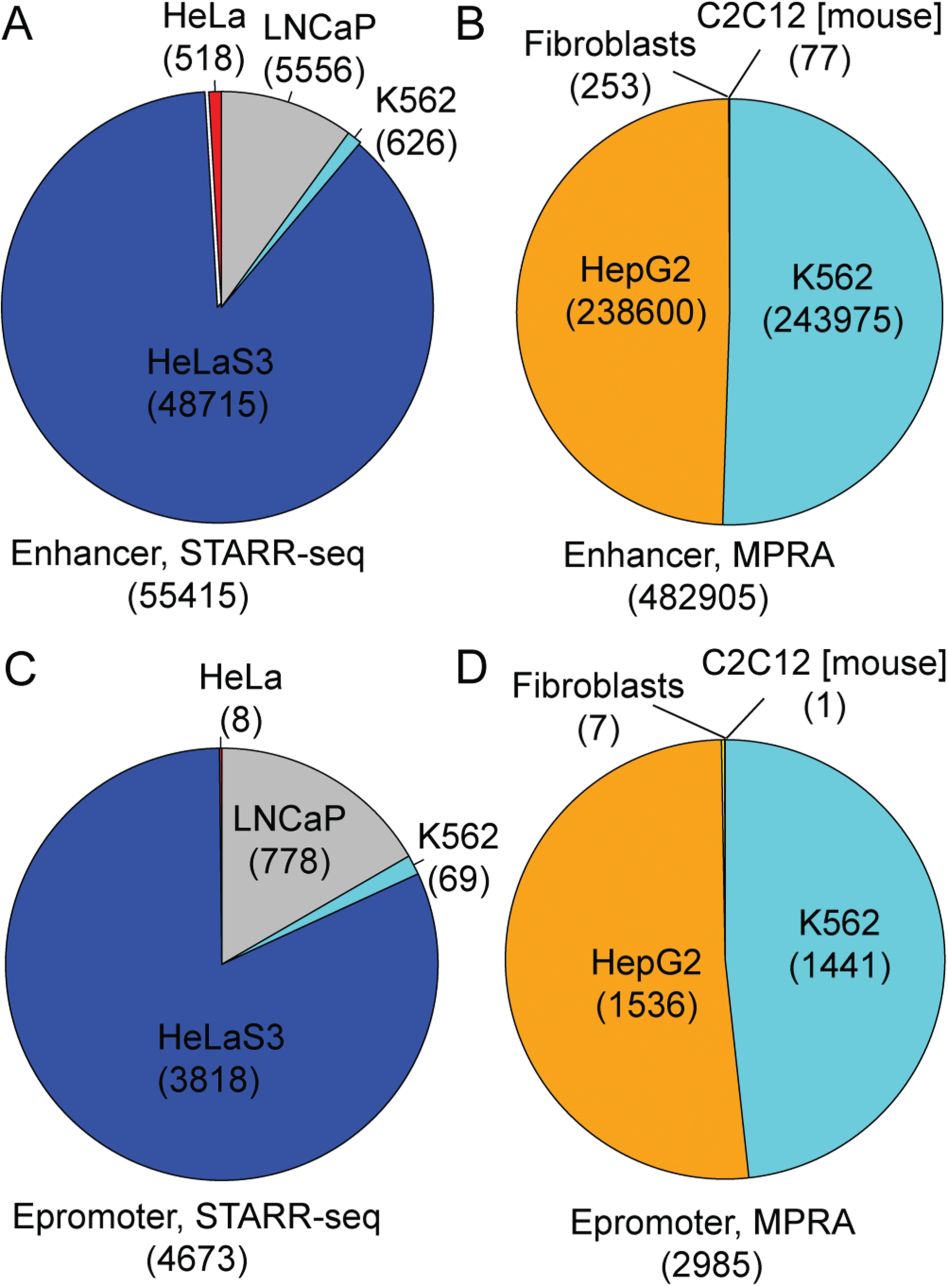
Data distribution of enhancers and epromoters by cell line and by methods.

RAEdb contains 538 320 enhancers derived from eight studies; 55 415 and 482 905 of these enhancers were derived from data generated by STARR-seq and MPRA method (Figure 2A and B), respectively. All the enhancers generated by STARR-seq belong to four human cell lines. Over three-fourth of them were found in HeLaS3 cell line, then the LNCaP, K562 and HeLa cell line. The enhancers generated by MPRA mostly belong to two human cell lines, i.e. HepG2 and K562. In addition, there are a few enhancers belonging to a human cell line (Fibroblasts) and a mouse cell line (C2C12). Besides for enhancers, RAEdb also includes 7658 epromoters; 4673 and 2985 epromoters were identified from data generated by SATRR-seq and MPRA method, respectively. The cell line distribution of epromoters is similar to that for enhancers (Figure 2C and D).

### Usage and access

**Figure 3:**
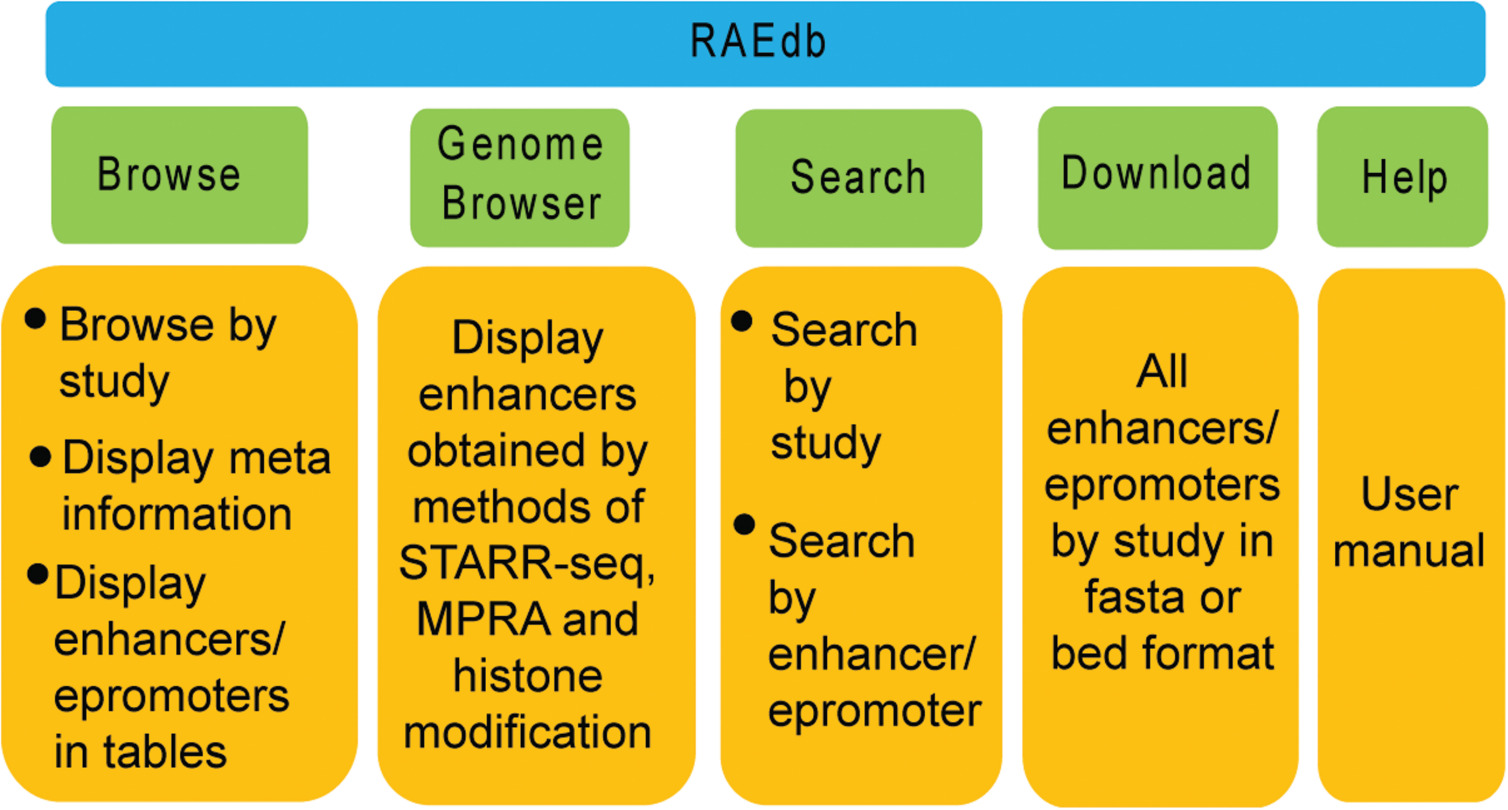
Structure of RAEdb database.

The RAEdb mainly includes Home, Browse, Genome Browser, Search, Download, Help and Contact Us pages (Figure 3).

**Figure 4:**
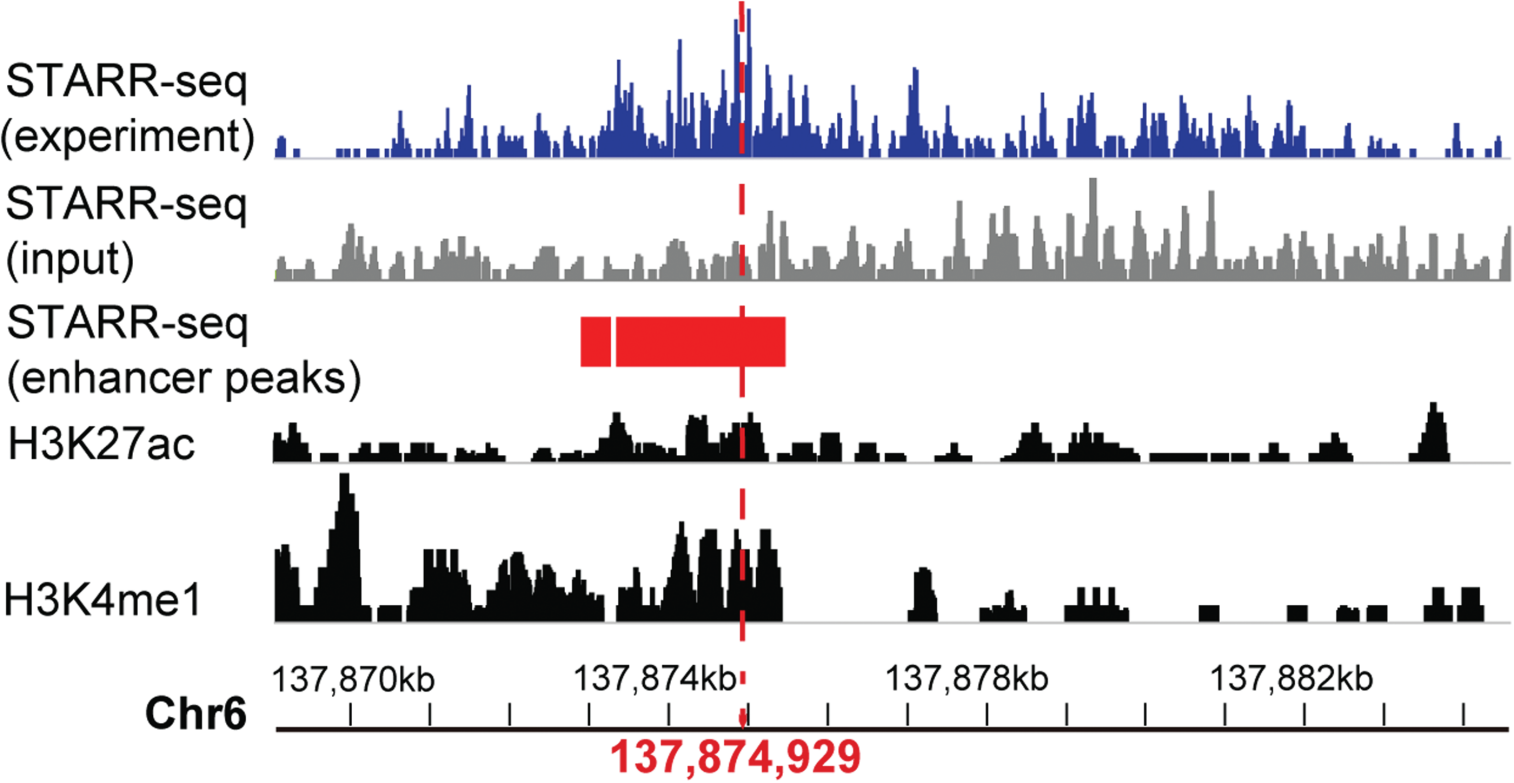
A systemic lupus erythematosus associated enhancer displayed in genome browser of RAEdb. It shows the activity measured by STARR-seq (two upper panels, from the dataset of GSE100423 in NCBI GEO database) and histone modification (two bottom panels, from the dataset of GSE29611 in NCBI GEO database) methods. The enhancer peaks called based on STARR-seq data were highlighted in red bars. The red perpendicular line refers to the SNP (rs2230926) associated with systemic lupus erythematosus.

#### Browse

All enhancers and epromoters were organized by sample in studies. For each study, this page displays the meta-information of the study, including title and abstract, cell lines, treatment for samples, accession number of data in NCBI GEO database and all enhancers or epromoters identified in the study. All the enhancers or epromoters from a sample are displayed in a table. For each enhancer, RAEdb displays the start and end positions of it on the chromosome, the activity score, and its sequences; for each epromoter, besides the start and end positions of it and the sequence, RAEdb also displays the name and id of its target gene.

#### Genome Browser

To explore the distribution of enhancers on the human or mouse genome, RAEdb provides a genome browser to query and visualize all enhancers collected in the database. As a comparison, enhancers that were identified by histone modification methods were also presented for some common cell lines (such as K562 and HeLaS3) in the genome browser. Users can select multiple samples simultaneously for comparing the distribution of enhancers identified in different cell lines or by different methods.

#### Search

RAEdb provides two ways to search the database. The first is to search studies by keywords, including data type (enhancer or epromoter), species, cell line and study name. The output is directed to the selected studies in the Browse page. The second is to search enhancers or epromoters by start and end positions, activity score, sequence and target gene (for epromoters) in the search box of the table in the Browse page.

#### Download

All data in RAEdb is freely available for download in either fasta or bed format. They are organized by study, method, species and cell line.

### A case study

Here we show how to explore enhancers of interest. The enhancer ranging from 137 869 065 to 137 884 663 on chromosome 6 was reported to be associated with systemic lupus erythematosus (4), which is an autoimmune disease in which the immune system mistakenly attacks healthy tissues. We want to know the activity of this enhancer in different cell lines. On the Genome Browser page, we could search the region of this enhancer. The output would display the activity score of this enhancer in multiple cell lines identified in multiple studies. It shows that this enhancer shows activity only in HeLaS3 cell line (Figure 4). The STARR-seq method identified two adjoining regions (137 872 975–137 873 278 and 137 873 347–137 875 412) of 2.3 kb (highlighted in red rectangle) with enhancer activity within the enhancer. Moreover, a single-nucleotide polymorphism (SNP) (marked by a red perpendicular line) (4) associated with the systemic lupus erythematosus is also located within this small region. As a comparison, the histone modification method presented multiple regions with enhancer activity (two bottom panels), which makes it difficult to identify the precise region acting as an enhancer.

## Discussion

The high-throughput reporter assays, especially the STARR-seq method, were reported to be more accurate and quantitative in identifying enhancers than methods developed in previous studies. The number of studies using STARR-seq technique has been growing significantly in the recent years. There is an urgent need for a database collecting enhancers identified in these studies. Here we present RAEdb, the first database for hosting and analyzing these enhancers. The main functions of RAEdb include searching studies and enhancers/epromoters of interest and visualizing enhancers in a genome browser.

Owing to the increasing interest in enhancers, the STARR-seq technology will be applied to a broader set of species, cell lines and conditions, and more data will be released in future. RAEdb will be updated in a timely manner (every season) with new released data from public studies. Continuous efforts would be devoted to improve the database. It could help much in understanding enhancers for both experimental and computational biologists.

## Notes

**Database URL**: http://www.computationalbiology.cn/RAEdb/index.php
